# Synthesis and In Situ Application of a New Fluorescent Probe for Visual Detection of Copper(II) in Plant Roots

**DOI:** 10.3390/molecules30244783

**Published:** 2025-12-15

**Authors:** Dongyan Hu, Jiao Guan, Wengao Chen, Liushuang Zhang, Xingrong Fan, Guisu Zhou, Zhijuan Bao

**Affiliations:** 1 College of Tobacco Science, Yunnan Agricultural University, Kunming 650201, China; 2023067@ynau.edu.cn (D.H.); guanjiao03@163.com (J.G.); 1779978922@163.com (W.C.); 15912598585@163.com (L.Z.); 15187599607@126.com (X.F.); zhgs05@163.com (G.Z.); 2 School of Modern Agriculture, Honghe Vocational and Technical College, Mengzi 661199, China; 3 Bijie Branch of Guizhou Provincial Tobacco Company, Bijie 551700, China; 4 Yongping Branch, Dali Prefecture Tobacco Company, Dali 672600, China

**Keywords:** fluorescence probe, rhodamine derivatives, copper ions, detection, root imaging

## Abstract

A new rhodamine-based fluorescent probe (RDC, rhodamine-based derivative) was rationally designed and synthesized for the highly selective, sensitive, and quantitative detection of Cu^2+^. The probe demonstrated outstanding specificity toward Cu^2+^, even in the presence of competing metal ions (e.g., Al^3+^, Fe^3+^, Cr^3+^, Na^+^, and K^+^), exhibiting negligible interference and confirming its robust anti-interference capability. A spectroscopic analysis revealed that Cu^2+^ induced spirocyclic ring cleavage, resulting in a colorless-to-pink colorimetric transition and enhancement of the yellow–green fluorescence at 590 nm. Upon addition of Cu^2+^, the fluorescence spectrum showed a linear response in the concentration range of 0.4–20 μM, with a correlation coefficient (R^2^) of 0.9907 and the limit of detection (LOD) calculated to be 0.12 μM. Meanwhile, Job’s plot analysis verified that the binding stoichiometry between RDC and Cu^2+^ was 1:1. The probe exhibits rapid response kinetics (<5 min) and non-destructiveness properties, enabling in vivo imaging. Under stress conditions, Cu^2+^ accumulated predominantly in root tips (its primary target tissue), with the following distribution hierarchy: root tips > maturation zone epidermis > xylem vessels > cortical cell walls. In conclusion, RDC is a well-characterized, high-performance tool with high accuracy, excellent selectivity, and superior sensitivity for plant Cu^2+^ studies, and this work opens new technical avenues for rhodamine-based probes in plant physiology, environmental toxicity monitoring, and rational design of phytoremediation strategies.

## 1. Introduction

As essential micronutrients, copper(II) ions (Cu^2+^) serve as catalytic cores for pivotal enzymes (cytochrome oxidase and superoxide dismutase) that underpin critical physiological processes, such as energy metabolism and oxidative stress management [1]. However, excess Cu^2+^ participates in Fenton reactions to generate hydroxyl radicals and triggers lipid peroxidation, protein denaturation, and DNA damage, ultimately causing cytotoxicity and organ dysfunction [2,3]. This concentration-dependent shift from essentiality to toxicity makes cellular Cu^2+^ homeostasis a fundamental aspect of life activities [4].

In plants, the uptake, transport, and intracellular homeostasis of Cu^2+^ directly influence photosynthetic efficiency, respiratory chain function, and antioxidant defense systems [5]. Given the pivotal role of Cu^2+^ in mediating these core plant functions, precise spatiotemporal regulation of Cu^2+^ absorption and distribution is indispensable for maintaining plant growth, development, and stress adaptability [6,7]. More importantly, soil contamination can drive Cu^2+^ accumulation in plants, threatening crop yields and quality, while also posing health risks via dietary intake [8]. Existing studies demonstrate that plant roots maintain precise Cu^2+^ homeostasis via molecular mechanisms including the COPT family copper transporters [9]. Moreover, different plants exhibit significant differences in their tolerance to Cu^2+^ and root accumulation capacity [10]. Thus, precise monitoring of Cu^2+^ dynamic distribution in plant roots is particularly crucial for advancing plant physiological studies. Nevertheless, traditional plant Cu^2+^ detection techniques, including atomic emission spectroscopy (AES), atomic absorption spectroscopy (AAS), UV–vis spectrophotometry, inductively coupled plasma mass spectrometry (ICP-MS), electrochemical methods, and ion chromatography, are inherently destructive and preclude real-time observation of intact specimens [11,12,13]. While cameras, scanners, and X-ray computed tomography (CT), as well as magnetic resonance imaging (MRI), can visualize root morphology, they are often unsuitable for tracking specific ion fluxes at molecular resolution [14,15]. Furthermore, high-resolution methods capable of visualizing spatiotemporal Cu^2+^ dynamics, such as concentration gradients across root zones or rapid stress responses, remain lacking. In recent years, fluorescent probe technology has emerged as a pivotal tool for Cu^2+^ detection in biological and environmental systems, owing to its advantages of real-time data acquisition, high spatiotemporal resolution, minimal sample perturbation, and operational simplicity [16,17,18,19]. Notably, through chemical modifications tailored to target specific ions, and when coupled with confocal microscopy, this technology enables subcellular-level imaging. This offers a powerful strategy for investigating biological samples [20,21,22,23,24,25].

Among these, rhodamine-based probes are particularly valued for their long-wavelength excitation/emission, robust photostability, high molar absorptivity, superior fluorescence quantum yield, and unique fluorescence switching mechanism via spirolactam ring opening [26,27,28]. Prior to target recognition, the probe remains non-fluorescent and colorless due to its closed spirocyclic form. Target binding triggers the opening of the spirolactam ring, which forms an extended conjugated system and generates distinct colorimetric and fluorogenic signals for visual or spectroscopic detection, thus producing a clear “off-on” response [29,30,31]. This characteristic offers a natural advantage for the high-sensitivity detection of Cu^2+^ [32].

Kaviya et al. developed a Cu^2+^-selective chemosensor based on a rhodamine 6G derivative, demonstrating high sensitivity with a detection limit of 1.42 nM for fluorometric analysis and 107 nM for smartphone-based detection [33]. Chang et al. reported a novel near-infrared fluorescent probe incorporating a rhodamine-based framework with a large Stokes shift (100 nm), which enabled Cu^2+^ imaging both in vitro and in vivo [34]. Sun et al. designed a rhodamine B-based fluorescent probe to detect the Cu^2+^ with the help of laser scanning confocal microscope in maize roots [35]. Gao et al. also synthesized a new bis-spirocyclic rhodamine B derivative with a large Stokes shift (70 nm) for Cu^2+^, which was applied to image Cu^2+^ in HepG2 living cells and within the mitochondria of paulownia sprout root cells [36].

Despite widespread use for metal ions, few fluorescence probes succeed in root Cu^2+^ imaging due to several significant limitations. Some probes exhibit poor selectivity for other transition metal ions (e.g., Fe^3+^, Hg^2+^, Zn^2+^) and are easily affected by interfering ions in biological matrices. Others exhibit unsatisfactory imaging quality for the tissue scattering and phytochrome interference of plants. Critically, most probes are optimized for animal cells or in vitro analyses. Insufficient consideration of plant-specific aspects such as cell wall penetrability, cytosolic stability, and phytotoxicity limits reliable quantitative imaging across functional root zones of plants (the root cap, the elongation zone, and the meristematic zone) [37,38,39]. Thus, developing new Cu^2+^ probes with enhanced selectivity, sensitivity, and plant compatibility remains critical for advancing both biological research and environmental monitoring. To address these challenges, a new probe RDC was developed by conjugating a carbohydrazide moiety to the carboxyl group of rhodamine B. The electron-donating nitrogen atom within the carbohydrazide moiety, which engages in highly specific lone-pair coordination with Cu^2+^ to form stable complexes, endows the probe with enhanced Cu^2+^ selectivity, improved biocompatibility, and applicability for plant tissue imaging.

## 2. Results and Discussion

### 2.1. Design and Synthesis of RDC

**Figure 1:**
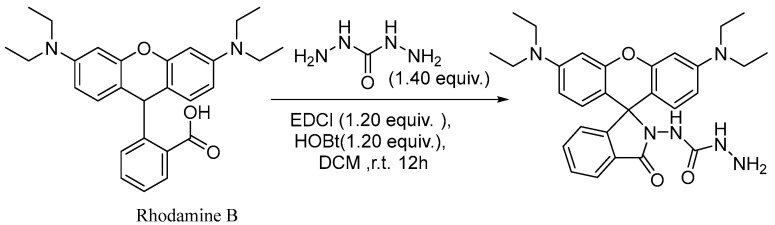
The synthesis of RDC.

In this work, rhodamine B was chosen as the fluorophore because of its good photochemical characteristics. The probe RDC was prepared by using a one-step condensation reaction (Figure 1). Rhodamine B (100 mg, 0.22 mmol) was dissolved in 10 mL of dichloromethane (DCM). Then, 1-(3-Dimethylaminopropyl)-3-ethylcarbodiimide hydrochloride (EDCl, 52 mg, 0.27 mmol), 1-hydroxybenzotriazole (HOBT, 37 mg, 0.27 mmol), carbohydrazide (28 mg, 0.31 mmol), and triethylamine (63 μL, 0.45 mmol) were added to the reaction solution. The mixture was stirred at room temperature overnight. The solvent was removed by evaporation under reduced pressure, and the residue was purified by silica gel column (PE/EA = 9:1, *v*/*v*) and thin-layer chromatography to obtain the compound RDC as a purple solid powder (54 mg, yield 50%). The final product was fully characterized by ^1^H NMR, ^13^C NMR, and HR-MS. ^1^H NMR (400 MHz, CDCl_3_) *δ* (ppm) = 8.03–7.95 (m, 1H), 7.56 (ddd, *J* = 14.3, 7.4, 1.3 Hz, 2H), 7.22 (d, *J* = 7.6 Hz, 1H), 6.48 (d, *J* = 8.9 Hz, 2H), 6.40 (d, *J* = 2.6 Hz, 2H), 6.32 (dd, *J* = 8.9, 2.6 Hz, 2H), 6.04 (m, 1H), 5.84 (s, 1H), 3.35 (q, *J* = 7.1 Hz, 8H), 1.17 (t, *J* = 7.0 Hz, 12H). ^13^C NMR (100 MHz, CDCl_3_): *δ* (ppm) = 12.58, 29.70, 44.42, 98.16, 104.20, 108.21, 123.54, 124.46, 128.11, 128.64, 129.23, 133.70, 150.73, 154.02, 159.09, 166.80. HR-MS, m/z: calculated [C_34_H_34_O_4_N_4_ +H]^+^ 515.2762, found 515.2761.

### 2.2. Spectral Properties of RDC and Selective Identification

**Figure 2:**
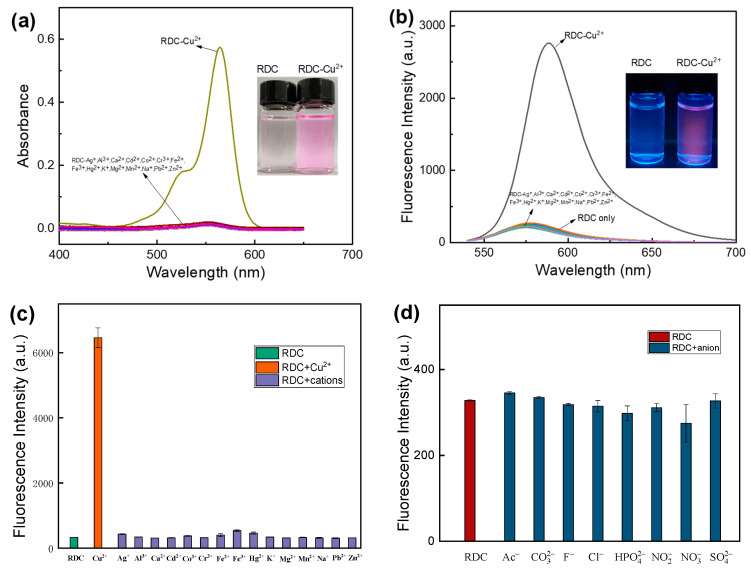
(**a**,**b**) Absorption and fluorescence spectra of probe RDC (10 μmol/L) in the presence of Cu^2+^ in MeOH/Tris-HCl buffer (pH =7.0, *v*/*v* =3/7, 50 mM); the fluorescence spectra were recorded with λ_ex_ = 520 nm. (**c**,**d**) Fluorescence intensity (λ_ex_/λ_em_ = 565/590 nm) for the effect of interfering cations and anions (10 μmol/L) on Cu^2+^ recognition by the probe.

To investigate the spectroscopic properties of RDC, the absorption and fluorescence spectra of its reaction with Cu^2+^ were measured. As shown in Figure 2a,b, RDC exhibited only weak fluorescence emission and negligible absorption in the visible region, consistent with its closed spirolactam structure. Upon addition of Cu^2+^, RDC underwent a distinct color change from colorless to pink, with a maximum absorption at 565 nm (ε = 5.16 × 10^4^ M^−1^cm^−1^). Concomitantly, the probe emitted bright yellow–green fluorescence, with strong emission at 590 nm.

To evaluate the selectivity of RDC for Cu^2+^, which is a critical criterion for its application in complex biological or environmental matrices, various coexisting metal ions (e.g., Cd^2+^, K^+^, Na^+^, Co^2+^, Ca^2+^, Fe^3+^, Al^3+^) and anions (e.g., Ac^−^, CO_3_^2−^, F^−^, Cl^−^, SO_4_^2−^) were individually added to the RDC probe solution (10 μmol/L) in 50 mmol/L Tris-HCl buffer (pH 7.0) containing 30% methanol. As shown in Figure 2c,d, the RDC with Cu^2+^ (10 μmol/L) produced strong fluorescence response, whereas the other ions at the same concentration did not show this behavior. Furthermore, the addition of a 100-fold molar excess of interfering metal ions to the RDC probe solution did not show any significant alterations in solution color or fluorescence intensity (Figure S1 in the Supplementary Information). Therefore, RDC demonstrated exceptional selectivity for Cu^2+^ over all other examined ions, enabling direct visual detection of Cu^2+^, even in the presence of competing species.

### 2.3. Optimization of Experimental Conditions

**Figure 3:**
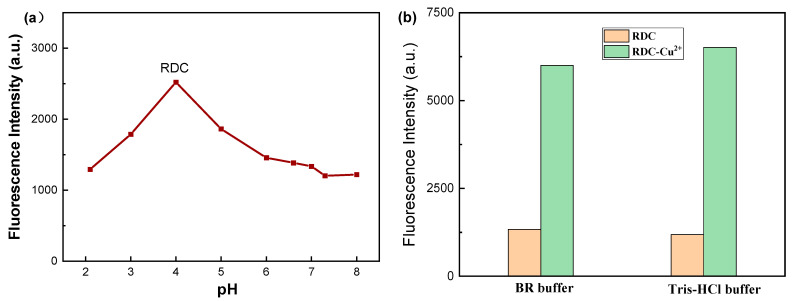
(**a**) The effects of the pH solution on the fluorescent efficiency of RDC (10 µmol/L); (**b**) the effects of the buffer solution on the fluorescent efficiency of RDC, λ_ex_/λ_em_ = 565/590 nm.

Various experimental conditions, including probe concentration, reaction media, and pH, were examined. The results showed that the response signals increased with the concentration of the probe. In this work, a 10 μmol/L probe was used as an appropriate concentration. Considering the application of the probe, the effect of pH on the absorption and fluorescence properties of RDC was studied. Without the addition of metal ions, the probe has neither obvious fluorescence changes nor dramatic color changes in the range of pH 6.0–8.0 (Figure 3a and Figure S2 in the Supplementary Information). However, when the pH value is lower than 5.0, the fluorescence intensity changes significantly, which is due to the ring opening and fluorescence release from the probe lactam due to strong protonation. When Cu^2+^ was added to the solution, the fluorescence intensity of the probe increased, accompanied by the color change, observable by the naked eye. Considering further study of the practical application of the probe in biological systems, the pH value was adjusted to medium. As shown in Figure 3b, the fluorescence intensity of both the probe and Cu^2+^ is significantly greater than that of the probe alone in two different buffer solutions at pH = 7.0. Furthermore, the ΔF(F-F_0_) value of the probe in the Tris-HCl buffer solution is higher than that in the BR buffer solution. Therefore, a 50 mmol/L Tris-HCl buffer solution at pH = 7.0 was selected as the medium for the test solution in the experiment.

Probe RDC can recognize Cu^2+^ in aqueous media containing 1% (*v*/*v*) DMSO (from the stock solution), while additional organic solvent markedly enhances its fluorescence intensity. With the increase in methanol fraction, the fluorescence signals showed a trend of increasing first and then decreasing (Figure S3 in the Supplementary Information). Therefore, in the test, 30% (*v*/*v*) methanol was used as a co-solvent. Meanwhile, upon mixing the probe with Cu^2+^, the solution underwent an immediate color change, confirming rapid reaction kinetics. Time course studies reveal that the fluorescence of the RDC-Cu^2+^ solution remained stable after 20 min and remained constant for at least 2 h (Figure S4 in the Supplementary Information).

### 2.4. Linearity and Binding Stoichiometric Ratio

**Figure 4:**
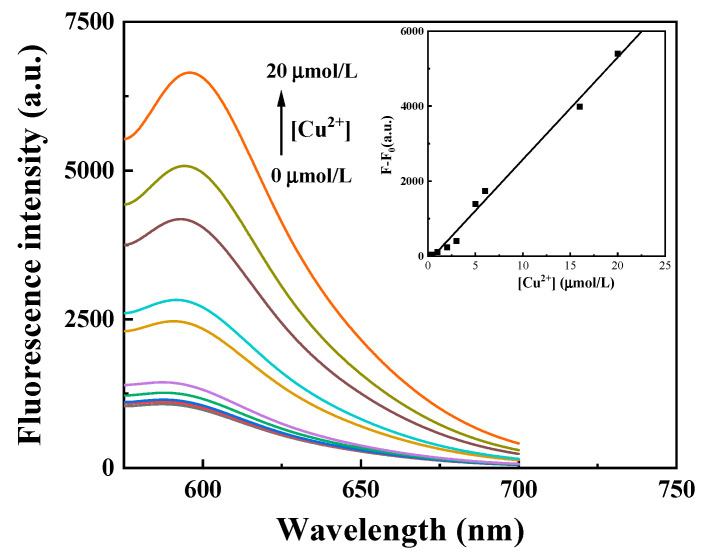
Fluorescence spectra of RDC–Cu^2+^ titration curves (Cu^2+^ concentration ranging from 0.4 to 20 μmol/L, CH_3_OH/Tris-HCl, 3/7, *v*/*v*, pH 7.0), at λ_ex_/λ_em_ = 565/590 nm. Color-coded lines represent varying concentrations of copper ions. The inset graphs show the linear relationships between RDC and Cu^2+^.

The fluorescence responses of probe RDC stock solutions were studied by adding different concentrations of Cu^2+^ at an excitation wavelength of 565 nm to investigate their effects on the fluorescence spectrum. As shown in Figure 4, there is a good linear relationship between the mixed solution of Cu^2+^ at different concentrations and the measured spectrum when the Cu^2+^ concentration ranges from 0.4 μmol/L to 20 μmol/L. The linear equation is ΔF = 272.51 × C(Cu^2+^) − 148.85 (R^2^ = 0.9907). The detection limit of the probe was calculated based on LOD = 3σ/k, where σ is the standard deviation of RDC blank response (*n* = 11), and K is the slope of the fitted line. According to the calculation, the detection limit of the probe for Cu^2+^ is 0.12 μmol/L.

**Figure 5:**
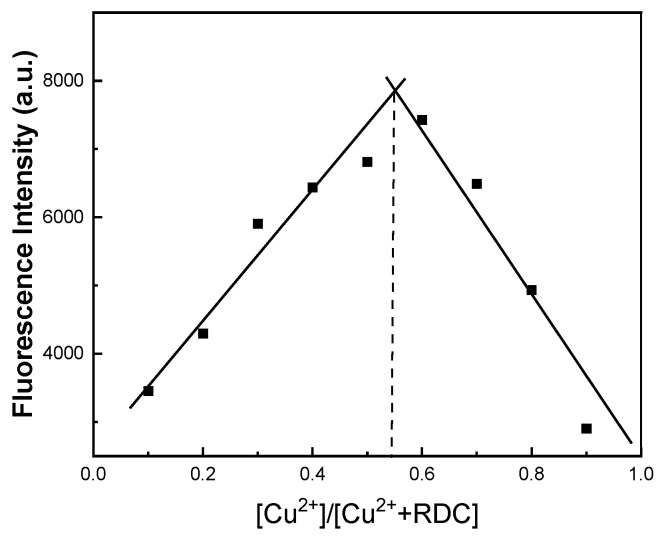
Job plot of RDC binding to Cu^2+^; the total concentration of RDC and Cu^2+^ is 20 μmol/L (λ_ex_/λ_em_ = 565/590 nm).

The combined stoichiometry of RDC and Cu^2+^ was determined by using the Job’s plot method. The total concentration of the solution was kept at 20 μmol/L, while the probe-to-Cu^2+^ ratio in the mixed solution was changed. The fluorescence intensities of RDC and Cu^2+^ were recorded at 590 nm, and the Job curve was plotted as shown in Figure 5. As can be seen from the figure, the fluorescence intensity of the mixture increased at first and then decreased with the increasing ratio of Cu^2+^. When the molar fraction of RDC detected Cu^2+^ at 0.55, the fluorescence intensity reached the maximum value. The results showed that the binding stoichiometric ratio of RDC + Cu^2+^ in aqueous solution was 1:1.

### 2.5. Fluorescence Reversibility of RDC and Reaction Mechanism

To fully understand the responses of RDC to Cu^2+^, further experiments were conducted to explore the reversibility of the corresponding processes. Ten eq of Cu^2+^ and EDTA were successively added to an aqueous solution of RDC (10 μmol/L), followed by repeated measurements of the fluorescence intensity. As shown in Figure S5 (Supplementary Information), weak fluorescence was observed in the initial solution, but the fluorescence intensity changed sharply after the addition of Cu^2+^, slightly increasing after the addition of EDTA. Following six cycles of alternating Cu^2+^ addition and EDTA chelation, with fluorescence intensity monitored during each cycle, the signal remained nearly unchanged. This confirms the irreversible binding between the probe and Cu^2+^.

**Figure 6:**
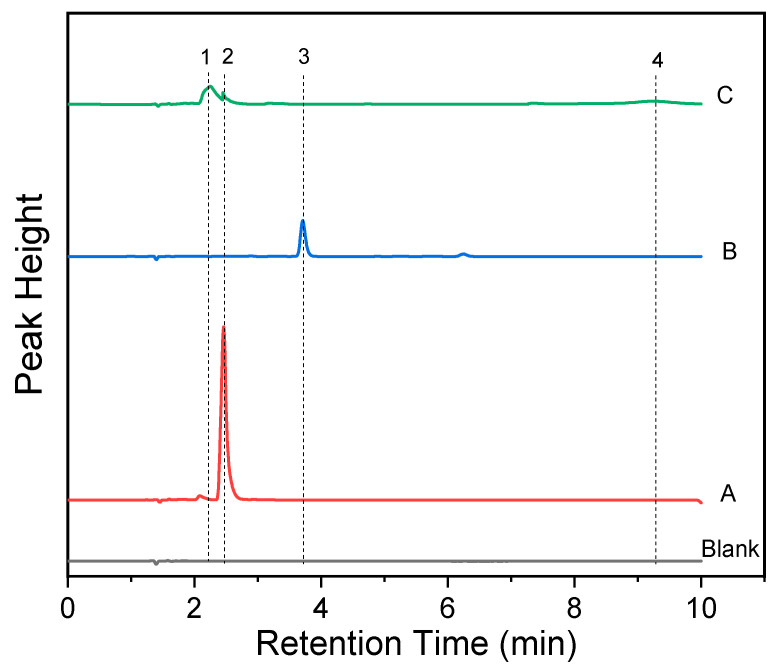
HPLC chromatograms of the reaction mechanisms of RDC to Cu^2+^. Blank: the reaction solution of 50 mmol/L Tris-HCl buffer (pH 7.0) containing 30% methanol; A: 20 μmol/L rhodamine B; C: 20 μmol/L RDC; D: the reaction products of 20 μmol/L RDC with 100 μmol/L Cu^2+^ according to the general procedure. The HPLC conditions: InertSustain AQ-C18w (4.6 mm × 150 mm) column, acetonitrile-1% acetic acid (65:35, *v*/*v*) as eluent (flow rate, 1.0 mL/min), 254 nm detection wavelength. The assignment of the peaks: (1) 2.25 min, an unidentified hydrolytic product (possibly the intermediate of Cu^2+^ complex); (2) 2.46 min, rhodamine B; (3) 3.71 min, probe RDC; (4) 9.24 min, unidentified products.

**Figure 7:**
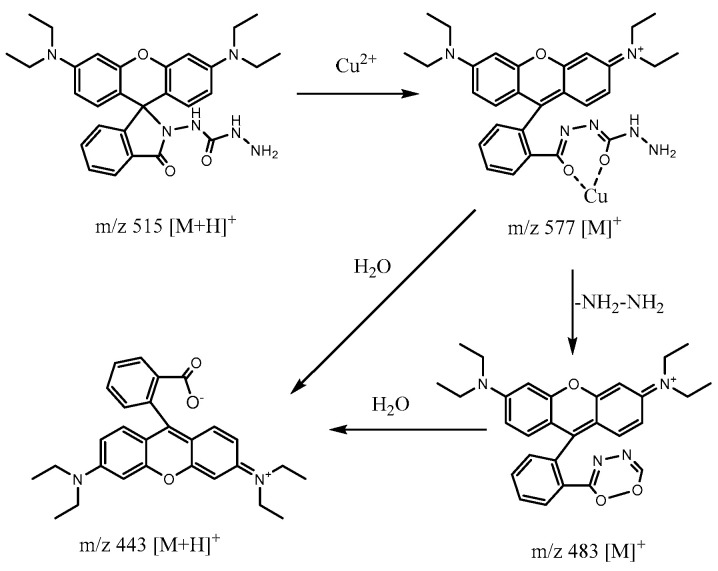
Possible reaction mechanisms of RDC to Cu^2+^.

In order to explore the reaction mechanism of the present system, the reaction products of probe RDC with Cu^2+^ were subjected to HPLC analysis. As shown in Figure 6, the chromatographic peaks of rhodamine B and RDC eluted at 2.46 min (peak 2 in curve A) and 3.71 min (peak 3 in curve B), respectively. Upon Cu^2+^ treatment, the RDC peak vanished, being replaced by three new peaks at 2.25 min, 2.46 min, and 9.24 min (curve C). Among these, the peak at 2.46 min corresponded to rhodamine B, confirming its formation in the reaction. To prove this, the reaction solution of RDC with Cu^2+^ was analyzed by using electrospray mass spectrometry (ESI-MS). The results revealed three primary ion peaks at *m*/*z* 443, 489, and 577, together with the RDC peak (*m*/*z* 515 [M + H]^+^) (Figure S6, Supplementary Information). The peak at *m*/*z* 443 was identified as rhodamine B, which confirms rhodamine B as the final reaction product. Despite unsuccessful attempts to isolate the species corresponding to the peaks at m/z 489 and 577, these two peaks are tentatively assigned to a Cu^2+^-RDC complex and a RDC intermediate, respectively, based on previous studies [40,41] and MS spectral analysis. The proposed fluorescence reaction mechanism is shown in Figure 7. The amide group of RDC first coordinates with Cu^2+^, followed by hydrolysis of the resulting copper complex. This process cleaves the amide bond and regenerates rhodamine B, thus restoring the fluorescence of the system.

### 2.6. Fluorescence Imaging of Cu^2+^ in Plant Seedling Root Tissue

**Figure 8:**
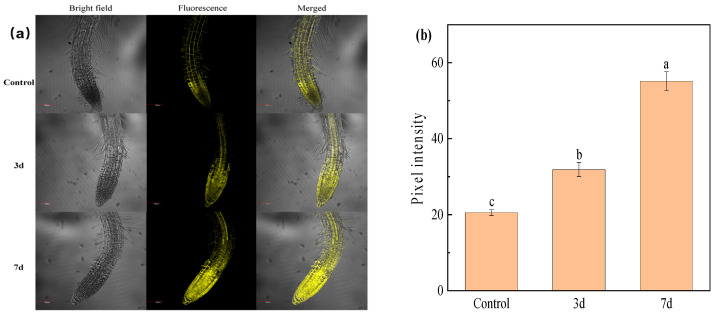
(**a**) Typical confocal (CLSM) images of Arabidopsis root tip region treated with Cu^2+^ stress (1 μg/mL) after incubation with a probe (10 μmol/L) (A: 0, B: 3d, C: 7d; eventually, the seedling age was 8d). Scale: 100 μm. (**b**) Quantitative analysis of the mean fluorescence intensity obtained from the corresponding fluorescence images. The different lowercase letters in the column indicate significant difference among treatments at *p* < 0.05 level.

**Figure 9:**
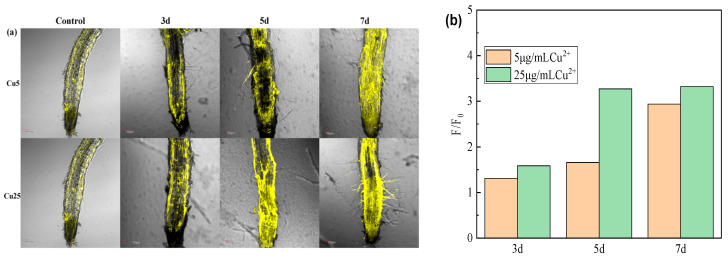

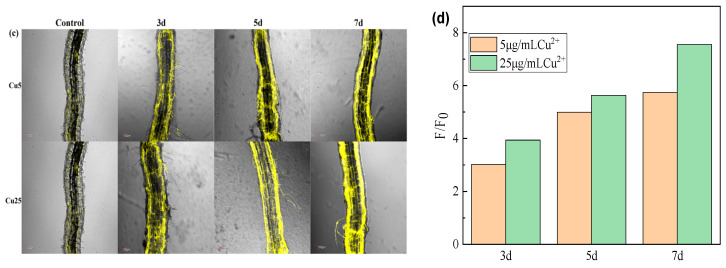
(**a**) Typical confocal (CLSM) images of the root tip area of burley tobacco treated with Cu^2+^ stress (5 μg/mL, 25 μg/mL) after incubation with a probe (10 μmol/L) (3d, 5d, and 7d indicate the stress duration of burley tobacco, and the final age of these seedlings was 8 days). Scale: 100 μm. (**b**) Normalized fluorescence intensity of images (**a**), in which F_0_ and F indicate the fluorescence intensity without and with Cu^2+^, respectively. (**c**) Typical confocal (CLSM) images of the mature zone of burley tobacco roots treated with different concentrations of Cu^2+^ after incubation with the RDC probe (10 μmol/L). Scale: 100 μm. (**d**) Normalized fluorescence intensity of images in (**c**), similar to (**b**).

Tobacco (*Nicotiana tabacum*) and Arabidopsis (*Arabidopsis thaliana*) are model species in plant physiology research. The former, characterized by a short growth cycle and a well-developed root system, serves as an ideal material for studying heavy metal uptake and translocation; the latter, with clear genomic information and a well-established genetic transformation system, provides a convenient platform for elucidating the molecular mechanism of Cu^2+^ metabolism [42,43]. Accordingly, this study employed synthetic probes to conduct imaging studies of copper ions in the seedling roots of Arabidopsis and tobacco. The results are presented in Figure 8 and Figure 9.

Copper uptake in plants is tightly regulated by metabolic pathways. The copper content in the root system is typically higher than that in above-ground tissues, with the highest accumulation specifically localized in root tips. Numerous studies have demonstrated that root tips serve as the primary target sites for copper, and roots exhibit the highest copper content among all plant tissues [44]. Furthermore, the elevated copper concentration in roots is localized to the root epidermis [45], with predominant accumulation in cell walls and vacuoles. Vessels—tubular structures in the xylem primarily responsible for water and mineral transport—mediate copper accumulation in xylem vessels. This phenomenon is attributed to the abundance of copper-chelating organic compounds in the xylem sap [46]. As illustrated in Figure 8a, owing to the intrinsic presence of endogenous Cu^2+^ in plants, the seedling roots of *Arabidopsis thaliana* that were not exposed to Cu^2+^ stress displayed weak fluorescence following incubation with the probe. Under Cu^2+^ stress, by contrast, Cu^2+^ was predominantly localized in the root tips of *A. thaliana*, followed by the epidermis of roots in the maturation zone, xylem vessels, and cortical cell walls (Figure 8a). This distribution pattern is consistent with previous findings. To provide a more intuitive visualization of the differences in fluorescent intensity, ImageJ 1.51j8 software was employed to quantify the relative fluorescent pixel intensity of each treatment group. As depicted in Figure 8b, following 3 and 7 days of Cu^2+^ stress exposure, the fluorescent intensity corresponding to Cu^2+^ in the root tips of *A. thaliana* increased by 55.07% and 168.42%, respectively.

Fluorescence imaging micrographs revealed distinct Cu^2+^ distribution patterns in tobacco seedlings root tissues (Figure 9a,c). In burley tobacco, characterized by a thicker root system, Cu^2+^ primarily accumulated within root tips, followed by cortical cell walls in the maturation zone. At a constant Cu^2+^ stress concentration, a fluorescence intensity analysis revealed a time-dependent increase across root tissues of both *A. thaliana* and burley tobacco as stress duration prolonged (Figure 8b and Figure 9b,d). Notably, at 25 μg/mL Cu^2+^, the rate of fluorescence intensity increases in root tips diminished after 5 days, indicative of Cu^2+^ accumulation approaching saturation within this tissue under high-concentration stress.

Furthermore, prolonged stress exposure (at equivalent durations) correlated with elevated Cu^2+^ levels in the roots as the concentration increased. This manifested as higher fluorescence intensity in both root tips and the maturation zone for both species. Crucially, a quantitative analysis (inferred from fluorescence intensity) demonstrated consistently higher Cu^2+^ enrichment within root tips compared to maturation zone cell walls.

**Figure 10:**
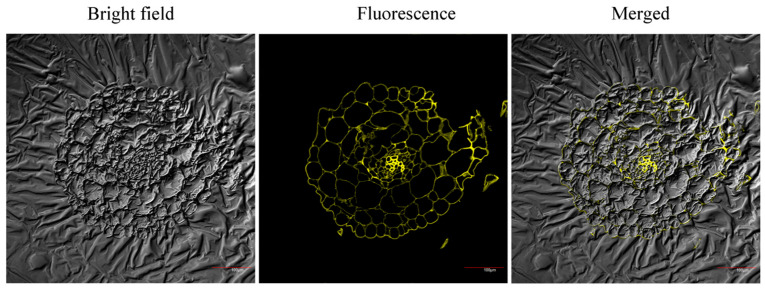
Confocal images of transverse sections of burley tobacco root tips treated with 25 μg/mL Cu^2+^ stress for 7 days after incubation with a probe (10 μmol/L). Scale: 100 μm.

To validate probe performance and inter-species consistency, cross-sectional imaging of burley tobacco roots was performed post-incubation (Figure 10). The observed Cu^2+^ (probe-detectable) localization mirrored patterns established in *A. thaliana*, predominantly confined to cell walls. This uniform distribution confirms the probe’s reliability and further underscores a common physiological response to excess Cu^2+^—preferential sequestration into root cell walls to mitigate cytotoxicity.

This study further assessed the cytotoxicity of the probe via the *MTS* assay. Experimental results demonstrated that the survival rate of lung cancer A-549 cells exhibited a dose-dependent decline with increasing probe concentrations. The inhibitory effect of 10 μM RDC against the proliferation of lung cancer A-549 cells cultured in vitro was 70.98 ± 1.29%. This phenomenon may be partially ascribed to the formation of complexes between the probe and copper ions in tumor tissues [47], which in turn impairs the viability of A-549 cells. This hypothesis warrants further experimental validation, thereby laying a theoretical foundation for the potential application of the probe in tumor suppression research.

## 3. Materials and Methods

### 3.1. Materials and Apparatus

Rhodamine and carbohydrazide were purchased from Aladdin Biochemical Technology Co., Ltd. (Shanghai, China). Other used reagents and solvents were obtained from commercial companies. All chemicals and solvents were used without further purification. UV-6100A spectrophotometer (Shanghai Metash instruments Co., Ltd., Shanghai, China) was used to measure UV–vis spectra. Fluorescence spectra were measured with Hitachi F-7000 fluorescence spectrophotometer (Tokyo, Japan). The ^1^H NMR and ^13^C NMR spectra were measured on a Bruker AMX 400 spectrometer with TMS as internal standard. Mass spectra were obtained from the Bruker solanX 70 FT-MS mass spectrometer (Billerica, MA, USA). The HPLC chromatograms of reaction solutions were measured using an Agilent-1260 infinity II chromatograph (Santa Clara, CA, USA). A Thermo Fisher (Waltham, MA, USA) Multiskan FC microplate reader was used for MTS assays. An Olympus (Tokyo, Japan) Fluoview FV1000 confocal microscope was used to record plant root imaging.

### 3.2. The Optical Spectra of Probe Toward Cu^2+^

The probe RDC stock solution (1 mmol/L) was prepared by dissolving the requisite amount in DMSO. Unless otherwise noted, all the tests were performed according to the following procedure. In a 5 mL colorimetric tube, 50 μL stock solution of RDC and 1.5 mL of methanol were mixed, followed by the addition of an appropriate Cu^2+^ sample solution and diluted with 0.05 mol/L Tris-HCl buffer (pH7.0 for spectra response, different pH for acidic effect) to a final volume of 5 mL. The reaction solution was shaken and the absorption and fluorescence spectra were recorded.

### 3.3. MTS Assays

In vitro cytotoxicity was evaluated in A549 lung cancer cells by MTS (Owen’s reagent) assay. The cells were cultured in 96-well plates with about 3000~5000 cells per well in the presence of 100 μL RMPI-1640 medium supplemented with 10% fetal bovine serum (FBS) under the condition of 37 °C and 5% CO_2_. The different concentrations of RDC (0, 1, 10 μmol/L) were added into the wells and incubated for 48 h. Then, 20 μL of MTS solution (1.90 mg/mL) and 100 μL of RMPI-1640 medium were added to each well and incubated for 4 h. The absorbance of the solution was measured at 492 nm by a microplate reader to calculate the cell viability.

### 3.4. Fluorescence Imaging of Cu^2+^ in Arabidopsis Thaliana and Burley Tobacco Root Tissues

*Arabidopsis thaliana* (wild type WT) and burley tobacco seeds were treated with 20%NaClO for 10~15 min, rinsed with sterilized water 3 times, and seeded on MS medium (*Arabidopsis thaliana*) and filter paper (burley tobacco) without copper, respectively. After incubating for 3 days, the seedlings were transplanted into nutrient solution containing different concentrations Cu^2+^ (0, 1, 5, 25 μg/mL) for 3~7 days. The roots of *Arabidopsis thaliana* and burley tobacco seedlings were collected and washed three times with 50 mmol/L Tris-HCl buffer (pH 7.0) to remove external copper and incubated with 10 µmol/L probe solution for 30 min at room temperature. Then, the root tissues were imaged using confocal laser scanning microscopy (CLSM) with an excitation wavelength of 565 nm.

## 4. Conclusions

In this study, a new rhodamine-based fluorescent probe was rationally designed and synthesized. In the absence of Cu^2+^, the probe exists as a colorless, non-fluorescent spirocyclic conformation. Upon Cu^2+^ coordination, the spirocyclic ring cleaves to form a conjugated structure, triggering a distinct colorimetric response and significant fluorescence enhancement. This enables both naked-eye visualization and quantitative detection of Cu^2+^ via fluorescence spectroscopy.

Notably, the probe enables direct in situ visualization of Cu^2+^ distribution and dynamic translocation, enabling the semi-quantitative visual analysis of Cu^2+^ in plants. In this test, the probe was successfully applied for in vivo fluorescence imaging of roots in *A. thaliana* and burley tobacco (*Nicotiana tabacum*). The imaging results and fluorescence intensity distribution were consistent with previously reported Cu^2+^ localization patterns, validating the probe’s high accuracy, excellent specificity, rapid response, and non-destructive properties for Cu^2+^ detection in living seedling roots. This work provides a robust tool for investigating plant Cu^2+^ metabolic pathways, guiding crop nutrient management, optimizing phytoremediation technologies, and evaluating ecological risks of soil heavy metal contamination. It also offers critical technical insights for advancing rhodamine-based probes in plant biology.

Compared with conventional heavy metal analysis methods, fluorescence probe-based imaging enables non-invasive detection of target ions in living samples. It facilitates precise delineation of ion localization, quantitative analysis of content fluctuations, and real-time tracking of dynamic translocation, making it a powerful tool for rapidly elucidating ion metabolic mechanisms in biological systems.

## Data Availability

Data is contained within the article and Supplementary Materials.

## Supplementary Materials

The following supporting information can be downloaded at https://www.mdpi.com/article/10.3390/molecules30244783/s1.

## Abbreviations

The following abbreviations are used in this manuscript:

Cu^2+^	Copper(II) ions
AES	Atomic emission spectroscopy
AAS	Atomic absorption spectroscopy
ICP-MS	Inductively coupled plasma mass spectrometry
CT	Computed tomography
MRI	Magnetic resonance imaging
NIR	Near-infrared
RDC	Rhodamine derivative
DCM	Dichloromethane
EDCl	1-(3-Dimethylaminopropyl)-3-ethylcarbodiimide hydrochloride
HOBT	1-hydroxybenzotriazole
HPLC	High Performance Liquid Chromatography
