# Supplementary material for: Synthesis and In Situ Application of a New Fluorescent Probe for Visual Detection of Copper(II) in Plant Roots
Source: Molecules. 2025 Dec 15;30(24):4783. doi: 10.3390/molecules30244783 (PMC12735961; doi:10.3390/molecules30244783)
Supplement: Supplementary file 1 [file molecules-30-04783-s001.zip › molecules-3990724-supplementary.pdf]

Synthesis and In Situ Application of a New Fluorescent Probe for Visual Detection of Copper(II) in Plant Roots

Dongyan Hu [1,†], Jiao Guan [1,2,†], Wengao Chen [1], Liushuang Zhang [1,3], Xingrong Fan [1,4], Guisu Zhou [1] and Zhijuan Bao [1,*]

[1] College of Tobacco Science, Yunnan Agricultural University, Kunming 650201, China;
2023067@ynau.edu.cn (D.H.); guanjiao03@163.com (J.G.);
1779978922@163.com (W.C.); 15912598585@163.com (L.Z.);
15187599607@126.com (X.F.); zhgs05@163.com (G.Z.)
[2] School of Modern Agriculture, Honghe Vocational and Technical College, Mengzi 661199, China
[3] Bijie Branch of Guizhou Provincial Tobacco Company, Bijie 551700, China
[4] Yongping Branch, Dali Prefecture Tobacco Company, Dali 672600, China
* Correspondence: baozhijuan@aliyun.com
† These authors contributed equally to this work.

Supplementary information

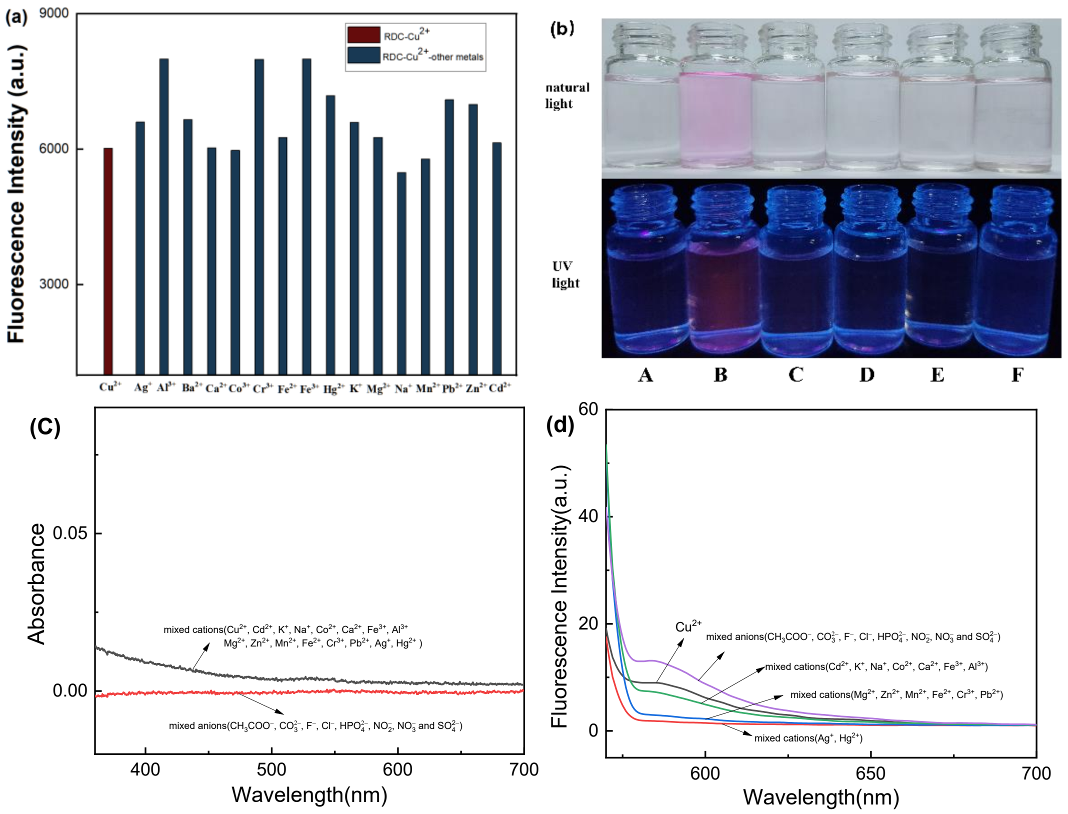

Figure S1. (a) Fluorescence spectra for the effect of interfering ions (1 mM) on Cu^{2+} (10 μM) recognition by the probe (10 μM). The fluorescence intensity was measured with λex/λem = 565/590 nm. **(b)** A picture of color reactions of RDC (10 μM) with various cations. A, RDC only; B, RDC + Cu^{2+} (10 μM); C, RDC + mixed ions (1 mM of Cd^{2+}, K^{+}, Na^{+}, Co^{2+}, Ca^{2+}, Fe^{3+}, Al^{3+}); D, RDC + mixed ions (1 mM of Mg^{2+}, Zn^{2+}, Mn^{2+}, Fe^{2+}); E, RDC + Cr^{3+}(1 mM); F, RDC + Pb^{2+}(1 mM). （**c, d**）Absorption and fluorescence spectra of cations and anions (1mM).

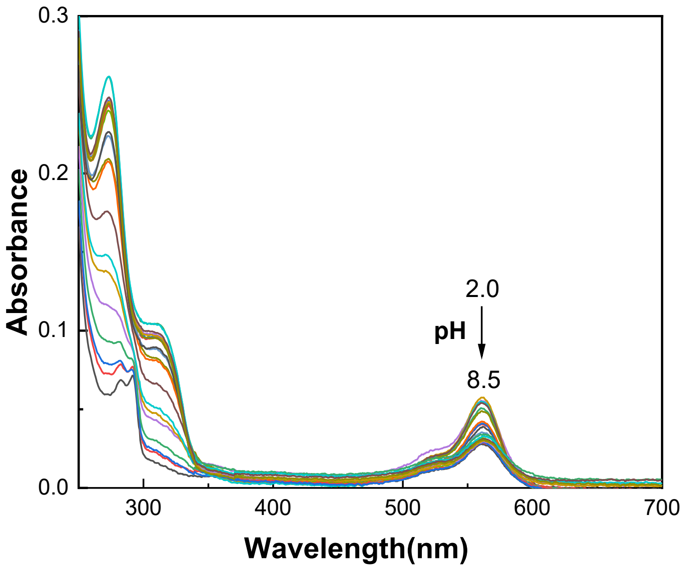

Figure S2. Absorption spectra of probe RDC (10 μM) in 0.04 mol/L BR buffer with different pH values from 2 to 8.5.

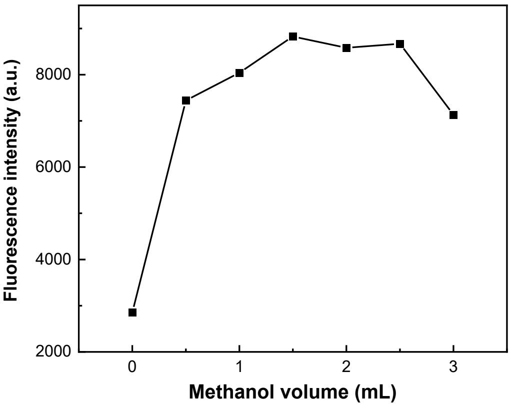

Figure S3. The effects of methanol volume on fluorescence intensity ($\lambda_{ex}/\lambda_{em}$ = 565/590 nm) of probe RDC+ Cu^{2+} (10 μM) in Tris-HCl buffer (pH =7.0, 50 mM) at room temperature.

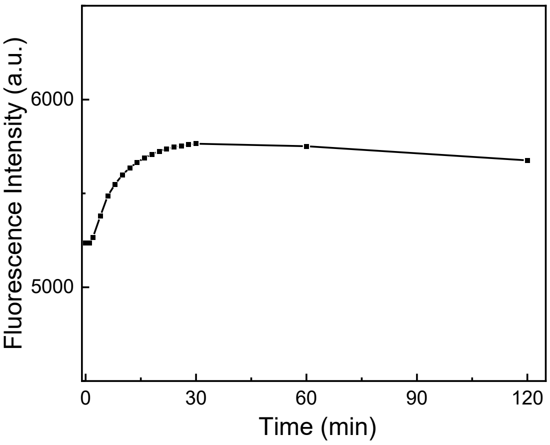

Figure S4. The change of fluorescence intensity ($\lambda_{ex}/\lambda_{em}$ = 565/590 nm) of probe RDC (10 μM) with time in presence of Cu^{2+} (10 μM) in MeOH/Tris-HCl buffer (v/v =3/7, pH =7.0, 50 mM) at room temperature.

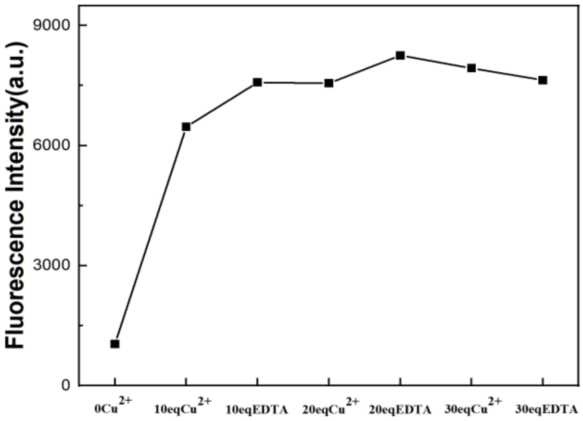

Figure S5. Fluorescence intensity changes after additions of Cu^{2+} and EDTA in RDC solution.

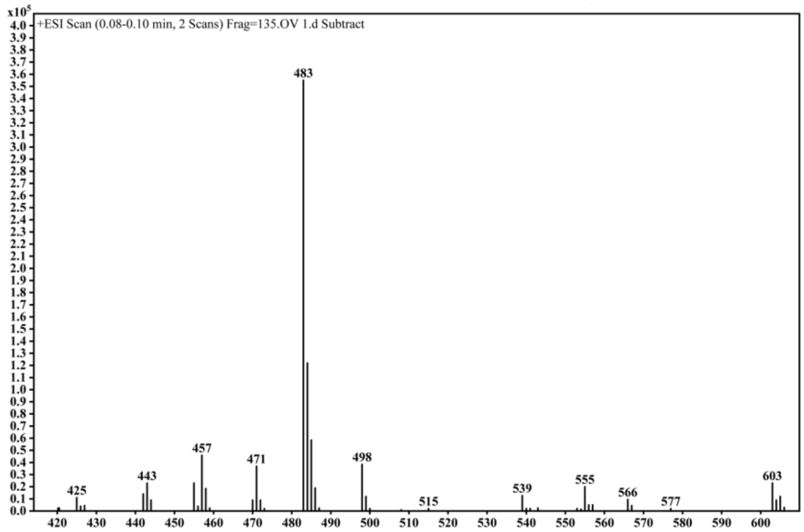

Figure S6. ESI mass spectrum of the reaction products of RDC with 1eq Cu^{2+} after 30 min.

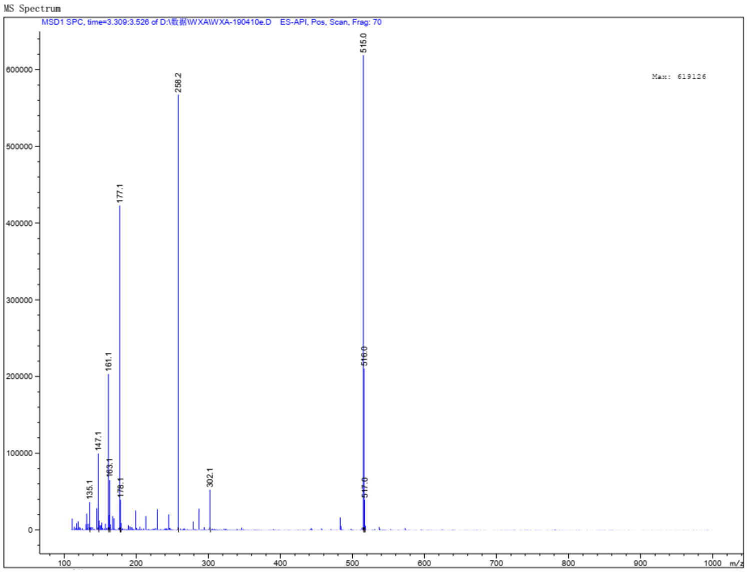

Figure S7. ESI mass spectrum of RDC

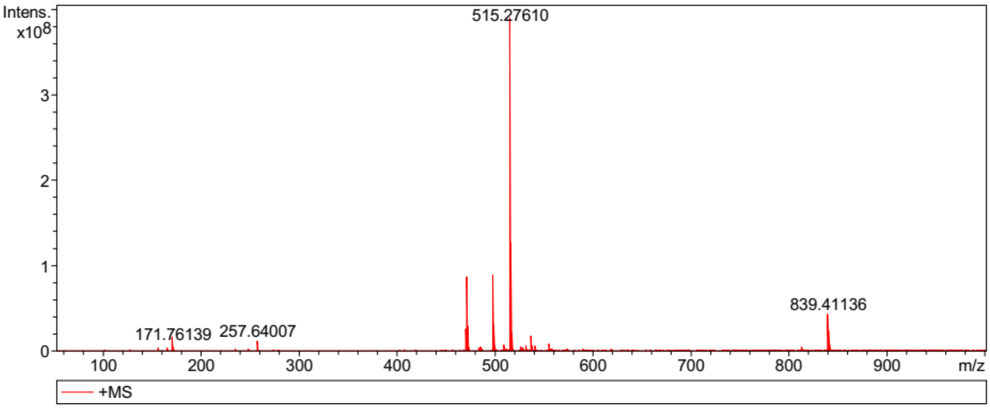

Figure S8. HR-MS of RDC

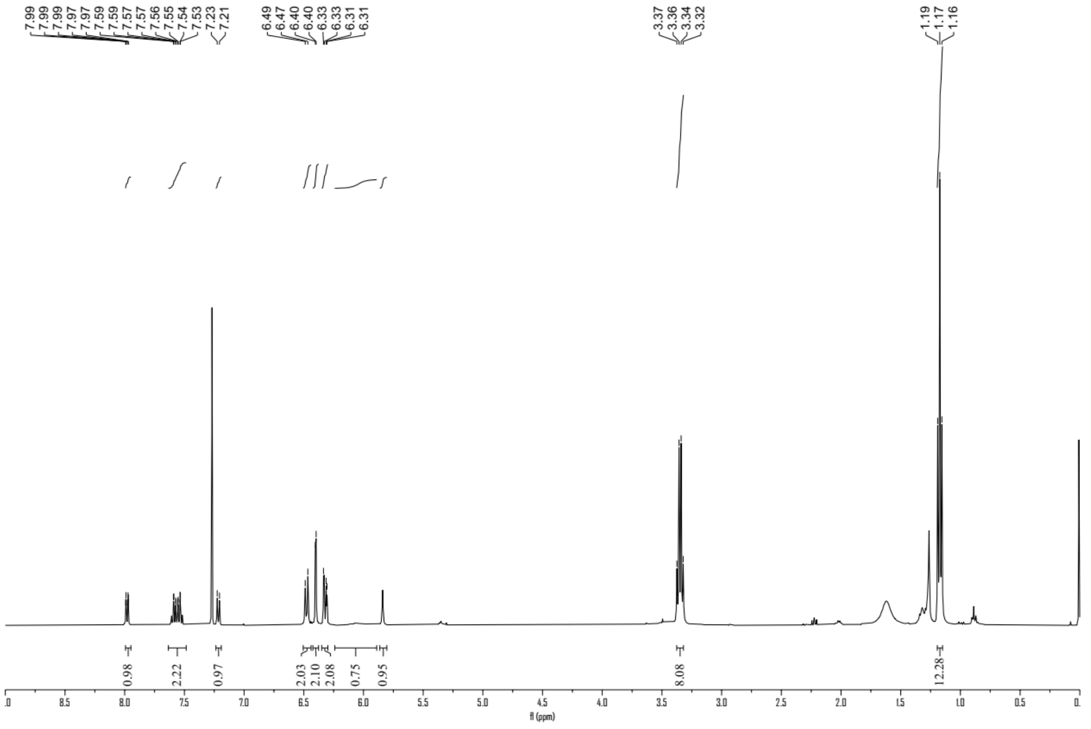

Figure S9. ^{1}HNMR (400 MHz, Chloroform-*d*) spectrum of RDC

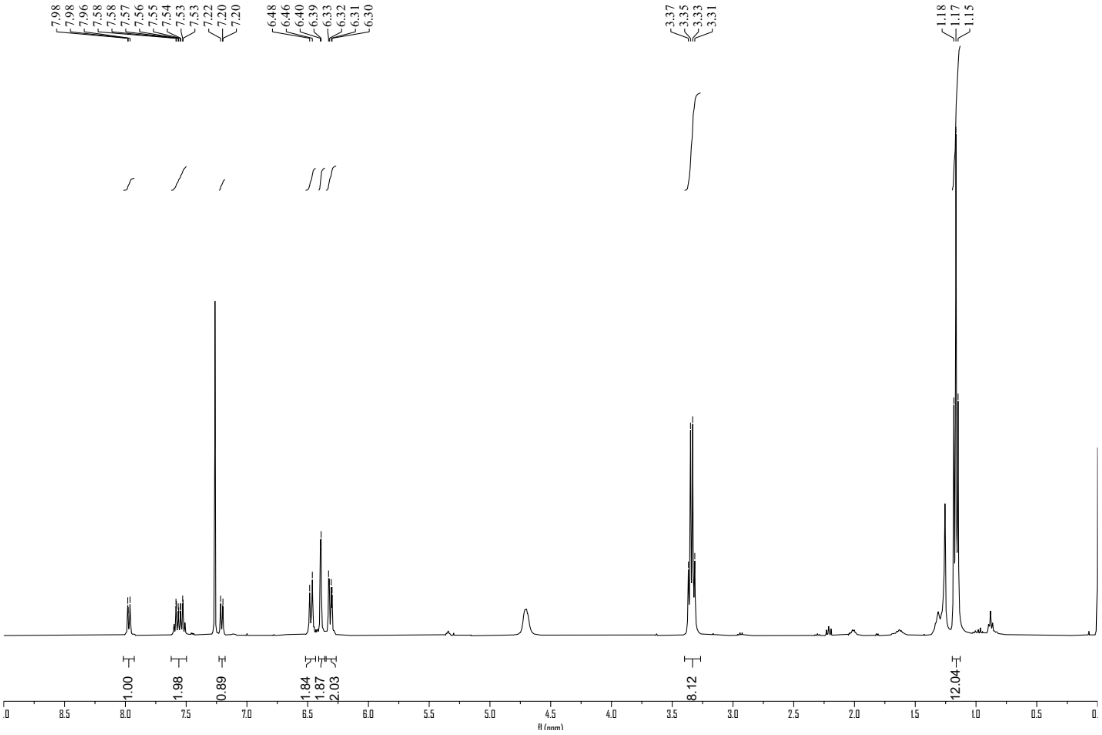

Figure S10. ^{1}H NMR (400 MHz, Chloroform-*d*) spectrum of RDC with D_2O.

^{1}H NMR: δ 8.0 (d, *J* = 7.4 Hz, 1H), 7.6 (ddd, *J* = 14.4, 7.4, 1.3 Hz, 2H), 7.2 – 7.0 (m, 1H), 6.5 (d, *J* = 8.8 Hz, 2H), 6.4 (d, *J* = 2.6 Hz, 2H), 6.3 (dd, *J* = 8.9, 2.7 Hz, 2H), 3.3 (q, *J* = 7.1 Hz, 8H), 1.2 (t, *J* = 7.0 Hz, 12H).

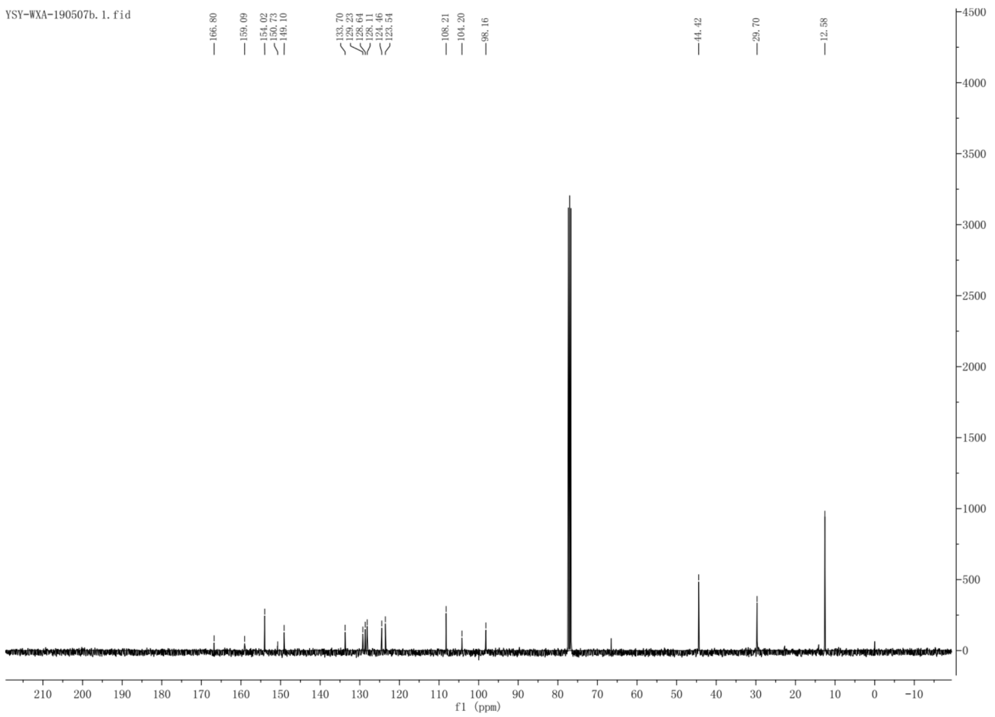

Figure S11. ^{13}C NMR (125 MHz, Chloroform-*d*) spectrum of RDC

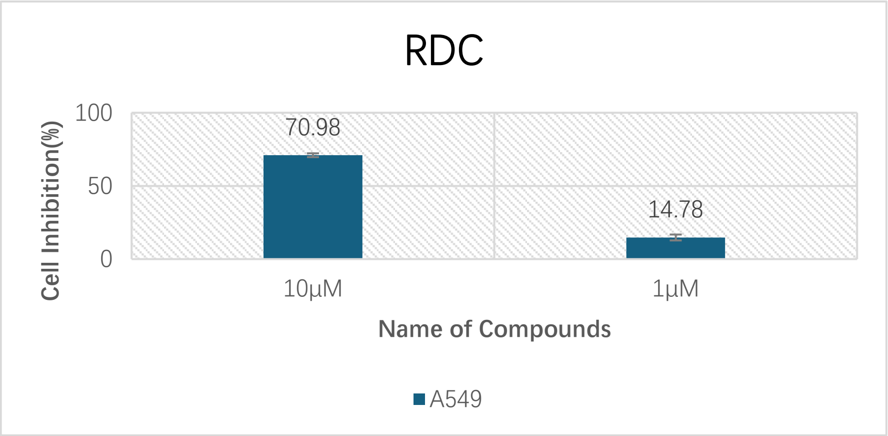

Figure S12. Cytotoxicity of RDC against lung cancer A-549 cells
